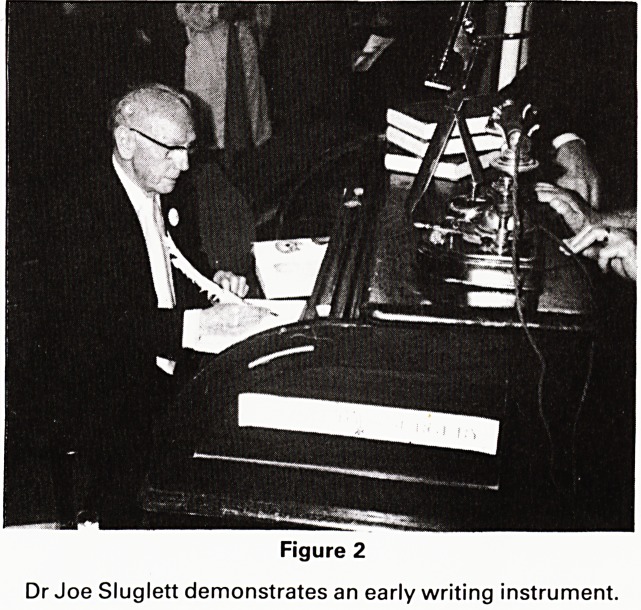# Opening of the Monica Britton Exhibition Hall

**Published:** 1986-02

**Authors:** M. G. Wilson


### Opening of the Monica Britton Exhibition Hall of Medical History

The first Exhibition Hall in this country to be devoted to Medical History was opened at Frenchay Hospital on Wednesday, October 30th, 1985. It was given by Mr J. H. Britton CBE MA LLD JP to Frenchay Health Authority in memory of his wife Monica.

In a short opening speech the Director of the project, Mr Colin Davidson paid tribute to the generosity and imagination of the donor and to the efforts of the project team and all the people who had worked so hard to get the opening exhibition together. The building is located adjacent to the Postgraduate Centre at Frenchay Hospital. The design is unusual and interesting, it is octagonal in shape and the interior is on two levels with a floor space of 1,200 sq. ft. Already, in the short time since the inception of the project many donations have enabled a permanent collection to be built up. Some items will be permanently on display but the programme will be changed from time to time to mount special exhibitions. Plans are already in hand for exhibitions on 'The History and development of Wound healing' Dec 2–4, 1985. 'Drug use and abuse', Spring 1986, and 'Bristol's contribution to the development of Anaesthesia' to coincide with the 2nd World Congress of Anaesthesia in July 1987. The opening exhibition featured the following topics—1. The development of the Obstetric Forceps—Mr J. Crossley. 2. Proprietary and 'Cure all' Patent Medicines—Mr S. J. Hamilton. 3. The Doctor's Surgery—Dr J. Sluglett. 4. How X-Rays began—Dr R. F. Harvey. 5. Prison Medicine and Forensic Psychiatry—Dr P. Trafford. 6. Homeopathic Medicine in Bristol—Dr D. Spence and Mrs S. Challis. 7. The quest to measure the pressure of the blood—Dr J. A. Bennet and Dr P. Hutton. 8. Cossham Hospital's Early Days—Mrs L. Morgan and Dr J. A. Bennett. 9. The Junker Chloroform Bottle—Dr T. N. P. Wilton. 10. The relief of pain in childbirth—Dr T. A. Thomas. 11. Materia Medica—Dr J. Alexander. 12. Early Surgical and Veterinary Instruments—Dr D. J. Warren. 13. Early surgical texts and instruments—Mr C. M. Davidson. 14. The deeds and seals of Frenchay Hospital—Mr R. Wheeler.

M. G. Wilson

**Figure 1**

Dr Edward Lace presents Mr Colin Davidson for the museum with a signed photograph of Joseph Lister, presented by him to a forbear of Dr Lace who was his house surgeon.

**Figure 2**

Dr Joe Sluglett demonstrates an early writing instrument.

## Figures and Tables

**Figure 1 f1:**
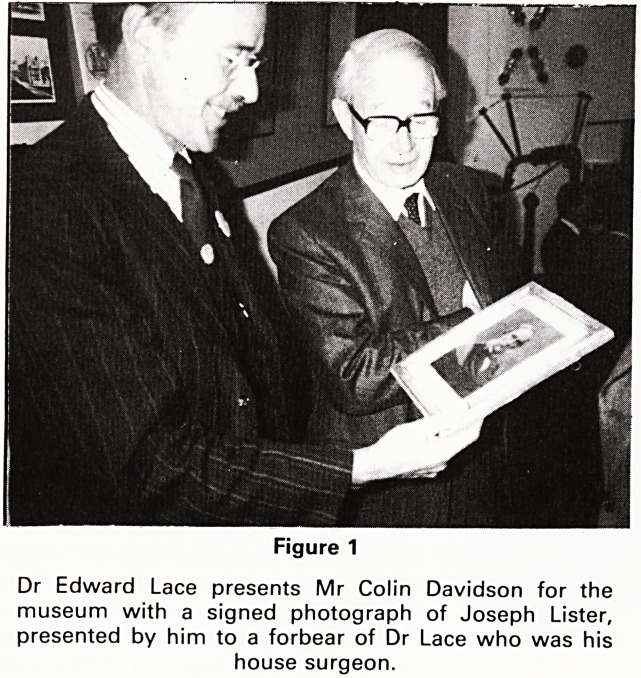


**Figure 2 f2:**